# Association of obesity with down-regulation of heat shock protein 40 expression and evidence that exercise retrieves its normal expression

**DOI:** 10.1186/1753-6561-6-S3-P1

**Published:** 2012-06-01

**Authors:** Jehad Abubakr, Mohamed Abu-Farha, Monira Al-Arouj, Fahad Al-Ghimlas, Irina Al-Khairi, Dalal Al-Mudhaf, Engin Baturcam, Abdullah Bennakhi, Preethi Cherian, Maha Hammad, Jeena John, Sina Kavalakatt, Abdelkrim Khadir, Ali Tiss, Samia Warsame, Said Dermime, Mohammed Dehbi

**Affiliations:** 1 Biomedical Research Department, Dasman Diabetes Institute, Kuwait, Kuwait

## Background

Obesity is a major risk factor for insulin resistance and diabetes. It is a metabolic disorder characterized by chronic-low grade inflammation and aberrant regulation of various forms of stress response in key metabolic sites. Heat shock response; a crucial host-defense mechanism against stressful conditions, is one of the key pathways that was shown to be deregulated in obesity-induced insulin resistance. Patients with T2D have reduced gene expression of Heat shock proteins (Hsps) which correlates with increased insulin resistance. Consistent with this, therapies that induce heat shock response such as heat therapy or physical exercise are associated with beneficial outcome. The objectives of this study are: 1) Investigate the expression pattern of Hsps and other chaperones and stress related proteins between lean and obese subjects, 2) Evaluate the effect of a defined exercise protocol on their expression pattern and 3) Correlate their expression with the inflammatory, metabolic and oxidative stress biomarkers.

## Materials and methods

A total of 600 human subjects divided into 3 equal groups using the body mass index as a reference (lean, overweight and obese) are being enrolled in a defined exercise protocol for 6 months. Blood samples and abdominal subcutaneous adipose biopsies were collected at 0, 3 and 6 months of the exercise. Gene expression profile was done by RT-PCR using RT-profiler-heat shock protein array kit consisting of 84 heat shock related genes. Cytokine and metabolic markers were investigated by multiplexing technology (Bioplex) using plasma samples. Markers of oxidative stress, namely ROS, and TBARS were measured on plasma or serum using commercially available kits. Western blotting and immunohistochemistry techniques were used to validate the gene expression data.

## Results

The analysis carried out on a first set of samples indicated differential expression of DNAJC5B, a member of Hsp-40 and crystallin alpha-B (CRYAB) genes between the lean and obese subjects. In obese subjects, we observed an upregulation (3.2 folds) of CRYAB gene expression. By contrast, the expression of DNAJC5B was down-regulated (>2 folds) in obese subjects in comparison to lean subjects. Interestingly, within 3 months of regular exercise, the levels of DNAJC5B in obese subjects were comparable to the lean subjects, suggesting that exercise can antagonize obesity-mediated DNAJC5B repression. The downregulation of DNAJC5B in obese subjects is associated with an increase in the inflammatory response such as TNF-α, IP-10 and MIP-1B. We also found a negative correlation between DNAJC5B expression and the release of metabolic and oxidative stress markers such as leptin, insulin, visfatin, ROS and TBARS.

## Conclusions

Our result demonstrated the differential expression of CRYAB and DNAJC5B genes in obese subjects in comparison to lean and highlighted their roles in obesity. Exercise was able to restore the aberrant expression of DNAJC5B gene and the existence of a negative correlation between DNAJC5B expression and the inflammatory, metabolic and stress responses is suggestive of a protective role of DNAJC5B and could therefore be a potential therapeutic target.

